# Engineering Soluble Recombinant BCMA for Ide‐Cel Labeling

**DOI:** 10.1002/eji.70251

**Published:** 2026-07-29

**Authors:** Ly Phuong Vy Nguyen, Christelle Oblet, Alice Barbarin, Louis Waeckel, Farah Kotaich, Clara Petit, Marine Mabily, Claire Carrion, André Herbelin, Jean Marc Gombert, Xavier Leleu, Emilie Chalayer, Murielle Roussel, Patrick Legembre

**Affiliations:** ^1^ INSERM U1262, UMR CNRS 7276 Université de Limoges Limoges France; ^2^ IRMETIST INSERM U1313 Université de Poitiers Poitiers France; ^3^ Inserm U1111, CNRS UMR 5308, CIRI Faculté de Médecine Jacques Lisfranc Saint‐Priest‐en‐Jarez France; ^4^ Immunology Laboratory CHU Saint‐Etienne Saint‐Etienne France; ^5^ Service D'immunologie et Inflammation CHU de Poitiers Poitiers France; ^6^ Service D'oncologie Hématologique et de Thérapie Cellulaire CHU de Poitiers Poitiers France; ^7^ Hématologie Clinique et Thérapie Cellulaire CHU Dupuytren Limoges France

**Keywords:** BCMA, CAR‐T, flow cytometry, molecular tool

## Abstract

Chimeric antigen receptor (CAR)‐T‐based strategies target the B‐cell maturation antigen (BCMA) expressed on the surface of malignant plasma cells in patients with multiple myeloma (MM). Idecabtagene vicleucel (ide‐cel), an anti‐BCMA CAR‐T therapy, has shown promising results in the treatment of relapsed or refractory multiple myeloma (R/RMM). Notably, the extent of CAR‐T cell expansion correlates with objective response rates and complete responses, indicating that monitoring CAR‐T cells is critical for predicting clinical outcomes in these patients. Although several tools are available to monitor CAR‐T cells in patient blood samples, they remain costly, prompting us to develop novel fluorescent and soluble BCMA reagents for CAR‐T cell labeling. BCMA is a type III transmembrane protein, and we found that a recombinant construct consisting of the BCMA extracellular domain fused to green fluorescent protein (GFP) failed to traffic through the Golgi apparatus and was not secreted. Incorporation of the interferon‐α2 signal peptide restored intracellular trafficking and enabled efficient secretion of soluble BCMA‐GFP and BCMA‐mCherry fusion proteins. These reagents specifically labeled anti‐BCMA CAR‐T cells. Overall, our results establish a cost‐effective method to generate soluble BCMA probes and provide accessible tools for monitoring CAR‐T cells in translational settings.

## Graphical abstract

This study describes the generation of a recombinant IFNα2‐BCMAext‐GFP probe, which enables sensitive detection of BCMA‐directed CAR‐T cells. The probe efficiently identifies idecabtagene vicleucel (ide‐cel) CAR‐T cells in patients with multiple myeloma and provides a simple alternative to current BCMA‐based detection reagents.

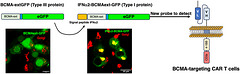

## 1 Introduction

Members of the tumor necrosis factor receptor superfamily (TNFRSF) are typically type I transmembrane proteins containing an N‐terminal signal peptide (SP) that directs protein trafficking through the endoplasmic reticulum (ER) and Golgi apparatus [1]. In contrast, B‐cell maturation antigen (BCMA; TNFRSF17) is a type III transmembrane protein that lacks an N‐terminal signal peptide [2]. BCMA binds two ligands, B‐cell activating factor (BAFF) and a proliferation‐inducing ligand (APRIL) [3], which regulate B‐cell activation, differentiation, and survival [4].

BCMA is a transmembrane receptor, which undergoes γ‐secretase‐mediated cleavage, generating a soluble form of BCMA (sBCMA) that acts as a decoy receptor for APRIL and BAFF [4, 5, 6]. Recent studies using two different BCMA knock‐out mouse models challenged the established role of membrane BCMA in plasma cell maintenance, demonstrating that long‐lived plasma cell survival is instead regulated by sBCMA, which limits APRIL availability through ligand sequestration [7]. Importantly, γ‐secretase‐generated sBCMA differs functionally from Fc‐fused recombinant BCMA proteins. Whereas recombinant BCMA‐Fc binds both APRIL and BAFF, naturally cleaved sBCMA exhibits preferential binding to APRIL [4], underscoring the importance of BCMA processing and structural conformation in determining ligand specificity.

BCMA is expressed on malignant plasma cells and has emerged as a major therapeutic target in multiple myeloma (MM) [1, 8]. Accordingly, several BCMA‐directed immunotherapies have been developed [9], including antibody‐drug conjugates, bispecific antibodies, and chimeric antigen receptor (CAR)‐T cell therapies [10]. Idecabtagene vicleucel (ide‐cel) is a BCMA‐targeting CAR, and ide‐cel CAR‐T cells represent an attractive therapeutic tool, inducing high response rates in patients with relapsed or refractory multiple myeloma [11, 12]. Clinical benefits are closely associated with CAR‐T cell expansion and persistence [11]. These observations emphasize the need for accurate and longitudinal monitoring of BCMA‐specific CAR‐T cells in patients.

Current approaches for CAR‐T cell detection include an antibody recognizing the Whitlow linker linear epitope connecting the ScFv heavy and light chains of ide‐cel [13, 14] and a recombinant BCMA fused to the Fc domain of IgG1 [14, 15]. These ide‐cel interactants need to be detected using a second step, including either a fluorochrome‐fused streptavidin or a fluorescent antibody [16]. While effective, BCMA‐Fc is costly and may affect BCMA conformation through Fc‐mediated dimerization.

In this study, we sought to develop soluble, fluorescent BCMA probes that preserve native binding properties while offering a cost‐effective and straightforward alternative for CAR‐T cell monitoring. We demonstrate that incorporation of a type I signal peptide (SP) is necessary to promote the efficient secretion of the BCMA extracellular domain fused to monomeric and fluorescent proteins, generating novel tools for labeling ide‐cel‐expressing CAR‐T cells in experimental settings and patients with MM.

## 2 Results and Discussion

### 2.1 Engineering a Soluble BCMA Construct

**FIGURE 1:**
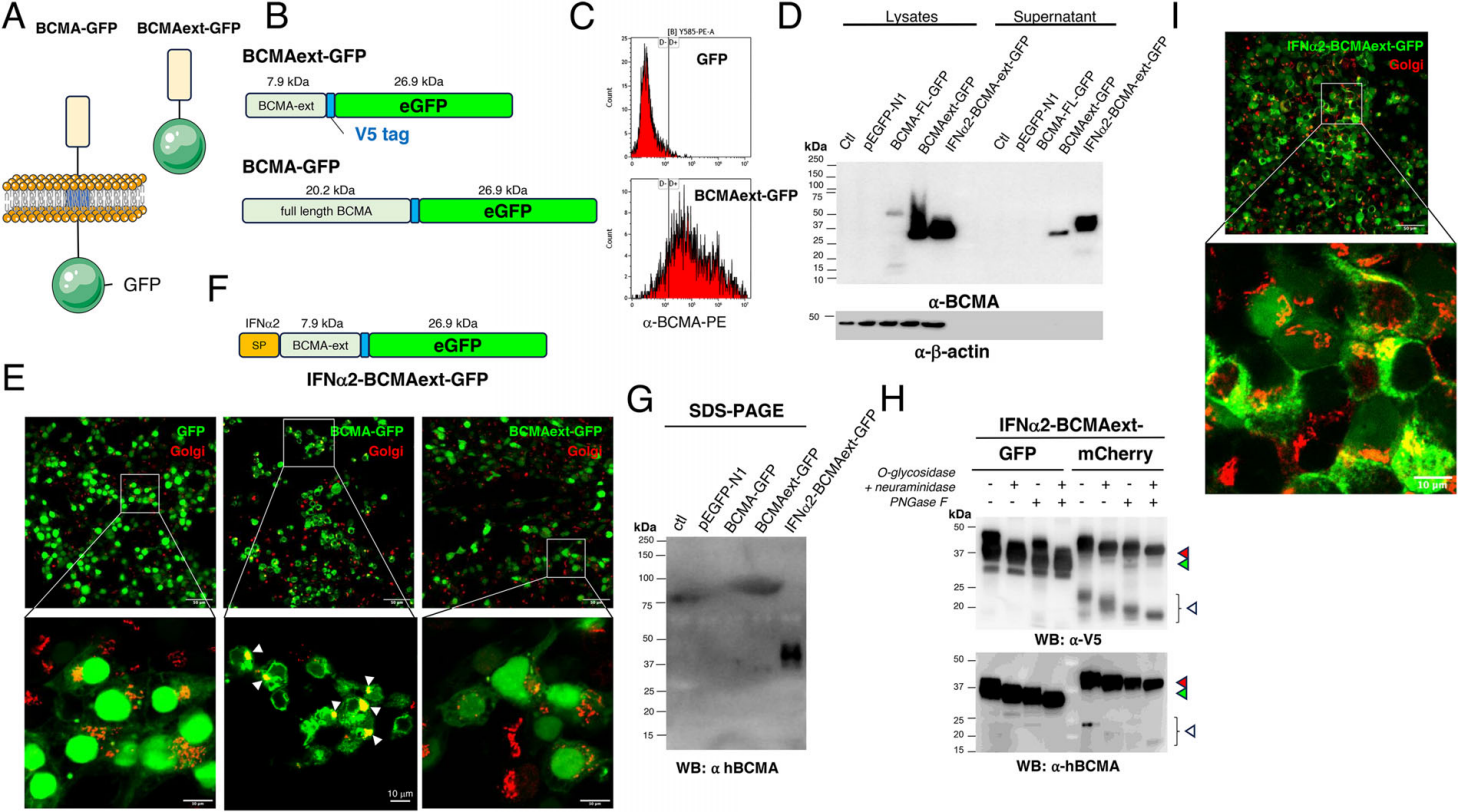
Generation of a soluble BCMA‐GFP construct. (A) Scheme of the BCMA‐GFP and BCMAext‐GFP proteins. The plasma membrane of the cell is depicted. (B) Description of the cDNA generated for the production of BCMA‐GFP and BCMAext‐GFP protein. The estimated molecular weight is reported. (C) HEK/293T cells were transfected with pEGFP‐N1 or full‐length BCMA‐GFP, and 24 h after transfection, cells were labelled using a PE‐conjugated anti‐BCMA mAb. The expression level of plasma membrane BCMA was analyzed by flow cytometry. Data are representative of three independently performed experiments. (D)HEK/293T cells were transfected with the indicated constructs, and after 5 days, cells were lysed, and supernatants were harvested. Cell lysates (60 µg) and supernatants (20 µL) were loaded in an SDS‐PAGE, and indicated immunoblots were performed. Data are representative of three independently performed experiments. (E) HEK/293T cells were transfected with the indicated constructs, and 48 h after transfection, confocal microscopy analyses were performed. Golgi was labelled using a mouse IgG1 anti‐GM130 followed by an AlexaFluor 647‐coupled goat anti‐mouse IgG1 antibody. Data are representative of three independently performed experiments. (F) Description of the cDNA generated for the production of IFNα2‐BCMA‐GFP. The estimated molecular weight is reported. (G) HEK/293T cells were transfected with the indicated constructs, and after 5 days, supernatants were harvested. Supernatants (20 µL) were loaded in an SDS‐PAGE, and an anti‐BCMA immunoblot was performed. A long‐time exposure of the immunoblot is depicted to reveal the BCMAext‐GFP construct expression. Data are representative of three independently performed experiments. (H) HEK/293T cells were transfected with the indicated constructs, and after 7 days, supernatants were harvested. Supernatants were subjected to anti‐V5‐mAb immunoprecipitation to purify V5‐tagged BCMA‐GFP and BCMA‐mCherry proteins. O‐glycosylation and N‐glycosylation were analyzed using deglycosylation enzymes following the manufacturer's instructions. N‐glycan removal from IFNα2‐BCMA‐GFP and IFNα2‐BCMA‐mCherry constructs was performed using the PNGase F glycan cleavage kit. O‐glycan removal was performed using O‐glycosidase combined with α‐2‐3,6,8,9 neuraminidase A. O‐ and N‐glycans were removed simultaneously using protein deglycosylation Mix II. IFNα2‐BCMA‐GFP and IFNα2‐BCMA‐mCherry constructs treated or untreated with glycohydrolases were subjected to western blot analysis. The green arrow indicates the untreated IFNα2‐BCMA‐GFP construct, while the red arrow shows the untreated IFNα2‐BCMA‐mCherry reagent. The white arrows depict degraded IFNα2‐BCMA‐mCherry products, and these constructs are labeled in the anti‐BCMA immunoblot. Upper panel: deglycosylation was analyzed by immunoblot using an anti‐V5 antibody. Lower panel: deglycosylation was analyzed by immunoblot using an anti‐human BCMA antibody. (I) HEK/293T cells were transfected with IFNα2‐BCMA‐GFP‐encoding vector, and 48 h after transfection, confocal microscopy analyses were performed. Golgi was labelled using a mouse IgG1 anti‐GM130 followed by an AlexaFluor 647‐coupled goat anti‐mouse IgG1 antibody. Data are representative of three independently performed experiments.

To develop a molecular tool for the detection of ide‐cel‐expressing CAR‐T cells, we first generated two BCMA‐based constructs. Full‐length human BCMA (amino acids 1–184) was fused in frame with a V5 epitope and enhanced green fluorescent protein (GFP), yielding BCMA‐GFP (Figure 1A,B). In parallel, we generated a truncated construct comprising only the extracellular domain of BCMA (amino acids 1–54) fused to V5 and GFP, designated BCMAext‐GFP (Figure 1A,B).

Flow cytometric analysis of HEK/293T cells transfected with BCMA‐GFP confirmed efficient expression of the chimeric protein at the plasma membrane (Figure 1C). Immunoblot analyses revealed that both BCMA‐GFP and BCMAext‐GFP were predominantly detected in cell lysates, with only trace amounts of BCMAext‐GFP recovered in culture supernatants (Figure 1D).

Confocal microscopy analyses showed that full‐length BCMA‐GFP trafficked through the Golgi apparatus before reaching the plasma membrane, whereas BCMAext‐GFP failed to colocalize with the Golgi marker GM130 (Golgi Matrix Protein of 130 kDa) and accumulated intracellularly, similar to GFP alone (Figure 1E). These observations indicate that the extracellular region of BCMA lacks an intrinsic signal for ER‐Golgi trafficking and secretion, consistent with its classification as a type III transmembrane protein lacking an N‐terminal signal peptide.

### 2.2 Signal Peptide‐Dependent Redirection of BCMA through the Secretory Pathway

We next sought to convert BCMA into a type I‐like secretory protein by adding an N‐terminal signal peptide. Based on previous optimization studies, we selected the human interferon‐α2 (IFNα2) SP, which efficiently promotes protein secretion in HEK/293T cells [17]. The IFNα2 SP was fused to the extracellular domain of BCMA to generate IFNα2‐BCMAext‐GFPq (Figure 1F).

Expression of IFNα2‐BCMAext‐GFP in HEK/293T cells resulted in a marked increase in protein secretion compared with the signal peptide‐deficient construct (Figure 1D,G). Notably, IFNα2‐BCMAext‐GFP migrated at a higher apparent molecular weight than BCMAext‐GFP under reducing conditions, despite similar predicted molecular masses (Figure 1B,F). Indeed, although both constructs had a predicted molecular mass of 32.8 kDa, BCMAext‐GFP migrated at a position below 37 kDa, and IFNα2‐BCMAext‐GFP migrated near 40 kDa (Figure 1D,G). Because BCMA contains a reported N‐glycosylation site at Asn42 [18], a modification that requires trafficking through the ER‐Golgi apparatus, we next examined whether glycosylation of IFNα2‐BCMAext‐GFP could account for the observed MW difference.

We performed deglycosylation assays to evaluate whether IFNα2‐BCMAext‐GFP carries N‐ and/or O‐linked glycans. For this purpose, IFNα2‐BCMAext‐GFP was purified using anti‐V5‐coated beads and treated with O‐glycosidase with Neuraminidase (to remove O‐glycans) and/or PNGase F (to remove N‐glycans) (Figure 1H). Each deglycosylation treatment reduced the apparent molecular mass of IFNα2‐BCMAext‐GFP (Figure 1H). Moreover, combined treatment with both deglycosylases produced an additive shift in molecular mass compared with either treatment alone (Figure 1H), indicating the presence of both N‐ and O‐linked glycans. Consistent with O‐glycosylation within the BCMA ectodomain, the NetOGlyc 4.0 prediction tool [19] identified Ser29 as a highly probable O‐glycosylation site.

Because glycosylation occurs in the ER and Golgi [20], confocal microscopy was used to examine the cellular distribution of IFNα2‐BCMAext‐GFP. These experiments revealed robust colocalization with the Golgi marker GM130 (Figure 1I). Together, these data demonstrate that the addition of an N‐terminal SP redirects the BCMA ectodomain to the secretory pathway and enables the production of a soluble, glycosylated BCMA extracellular domain.

### 2.3 Labeling of Ide‐Cel‐Expressing Cells with Soluble BCMA Probes

**FIGURE 2:**
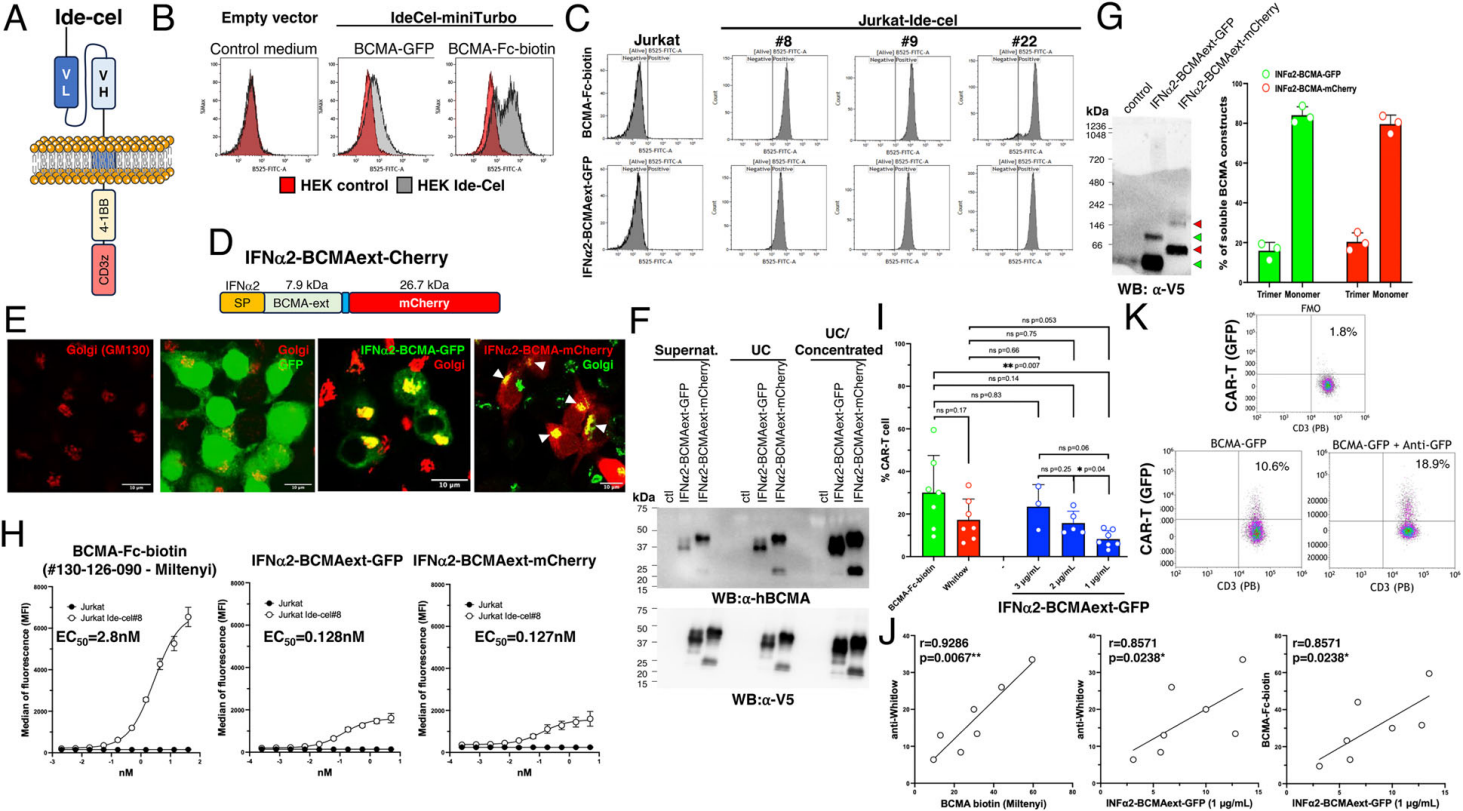
A new soluble BCMA to stain ide‐cel CAR. (A)Scheme of the ide‐cel CAR. The different domains of the light and heavy variable chain (VL and VH) are represented with the intracellular domains of 4‐1BB and CD3z proteins. (B) HEK/293T cells were transduced with the indicated lentiviruses, and 48 h after transduction, cells were labelled with BCMA‐biotin and revealed with an Alexa488‐conjugated streptavidin or with IFNα2‐BCMA‐GFP. The expression level of plasma membrane CAR ide‐cel was analyzed by flow cytometry. Data are representative of three independently performed experiments. (C) Jurkat cells were transduced with the indicated lentivirus and cloned by limiting dilutions. Clones were amplified and screened for ide‐cel CAR expression. The selected clones were labeled with BCMA‐Fc‐biotin/Alexa488‐conjugated streptavidin or with IFNα2‐BCMA‐GFP. Data are representative of three independently performed experiments. (D) Description of the cDNA generated for the production of IFNα2‐BCMA‐Cherry. The estimated molecular weight is reported. (E) HEK/293T cells were transfected with pEGFP‐N1, IFNα2‐BCMA‐GFP, or IFNα2‐BCMA‐Cherry, and after 48 h, cells were stained with the Golgi marker, GM130, using a secondary antibody conjugated with either an AlexaFluor 647‐coupled goat anti‐mouse IgG1 antibody or an AlexaFluor 488‐coupled goat anti‐mouse IgG1 antibody. Data are representative of three independently performed experiments. (F) HEK/293T cells were transfected with the indicated constructs, and after 7 days, supernatants were harvested. The raw, ultracentrifuged, and concentrated supernatants (20 µL) were loaded in an SDS‐PAGE, and anti‐BCMA and anti‐V5 immunoblots were performed. Data are representative of three independently performed experiments. (G) Left panel: ultracentrifuged and concentrated supernatants (20 µL) in F were loaded in a BN‐PAGE, and an anti‐V5 immunoblot was performed. Data are representative of three independently performed experiments. Right panel: for each soluble BCMA construct, a densitometric analysis was performed on the two bands observed in BN‐PAGE. Data are representative of three independently performed experiments. Data represent mean ± SD of three independent experiments. (H) The three ide‐cel‐expressing Jurkat clones shown in C were labelled with indicated concentrations of IFNα2‐BCMA‐Cherry, IFNα2‐BCMA‐GFP, or BCMA‐Fc‐Biotin (Miltenyi), and the mean of fluorescence (MFI) was assessed using flow cytometry. Data represent mean ± SD of three independently performed experiments. (I) CAR‐T cells were monitored in the blood of patients (between *n* = 3 and 7) using BCMA‐Fc‐Biotin or a whitlow antibody‐PE and compared with BCMA‐Fc‐GFP. The chart represents the percentage of positive CAR‐T cells in *n* = 3–7 patients with MM treated with ide‐cel CAR‐T cells. Data represent mean ± SD; *p < 0.05 and **p < 0.01, using two‐tailed Mann–Whitney test. (J) Correlations between the percentage of CAR‐T cells detected in the blood of patients with MM using IFNα2‐BCMA‐GFP (1 µg/mL), BCMA‐Fc‐Biotin, and an anti‐whitlow antibody‐PE. Correlation between each labeling was analyzed using a nonparametric Spearman correlation assay (*p < 0.05 and **p < 0.01). (K) Dot plots are representative of the IFNα2‐BCMA‐GFP staining with or without AF488‐conjugated anti‐GFP mAb. Fluorescence minus one (FMO) control is the sample that contains all the fluorophores in the multicolor panel except IFNα2‐BCMA‐GFP.

We next evaluated whether IFNα2‐BCMAext‐GFP could be used to detect ide‐cel‐expressing cells. HEK/293T cells transduced with an ide‐cel CAR construct (Figure 2A) were stained either with a commercial BCMA‐Fc‐biotin reagent or with IFNα2‐BCMAext‐GFP. Although IFNα2‐BCMAext‐GFP specifically labeled CAR‐expressing cells, the fluorescence intensity was lower than that obtained with the commercial reagent (Figure 2B).

To further assess labeling efficiency, stably ide‐cel‐expressing Jurkat T‐cell clones were generated by lentiviral transduction and limiting dilution (Figure 2C). All clones were stained by both BCMA‐Fc‐biotin and IFNα2‐BCMAext‐GFP, with consistently higher mean fluorescence intensity obtained using the biotinylated reagent (Figure 2C). This difference reflects either the weak fluorescence of GFP or the signal amplification provided by an indirect detection using an Alexa488‐conjugated streptavidin as a secondary reagent.

To further investigate this question, GFP was replaced by the red fluorescent protein mCherry, a derivative of the monomeric fluorescent protein mRFP1 originally isolated from Discosoma sp. and characterized by excellent fluorescence properties [19]. We generated an IFNα2‐BCMAext‐mCherry fusion protein (Figure 2D) and found that, similarly to its GFP counterpart, recombinant BCMA colocalized with the Golgi apparatus in transfected cells (Figure 2E). IFNα2‐BCMAext‐mCherry was also efficiently secreted into the culture supernatant and migrated on SDS‐PAGE at an apparent molecular weight below the 50 kDa marker (Figure 2F). Although the predicted molecular weights of IFNα2‐BCMAext‐mCherry and IFNα2‐BCMAext‐GFP were nearly identical (34.8 and 34.6 kDa, respectively), IFNα2‐BCMAext‐mCherry consistently exhibited a slightly higher apparent molecular weight than IFNα2‐BCMAext‐GFP under denaturing and reducing conditions (Figure 2F). Of note, a previous report also showed that mCherry displays a higher apparent molecular weight than eGFP [21]. The molecular basis of this discrepancy remains unclear and may involve differences in protein conformation and/or posttranslational modifications.

Similar to IFNα2‐BCMAext‐GFP, the mCherry fusion protein was modified by both N‐ and O‐linked glycosylation (Figure 1H). The anti‐V5 immunoblot revealed that a part of IFNα2‐BCMAext‐mCherry underwent a cleavage releasing a fragment sensitive to both O‐ and N‐glycosydase (Figure 1H). Because this fragment below 20 kDa was recognized by an anti‐BCMA antibody (Figure 1H), and this fragment was sensitive to both N‐ and O‐deglycosylations, we concluded that the extracellular region of BCMA was N‐ and O‐glycosylated.

We next investigated the stoichiometry of IFNα2‐BCMAext‐GFP and IFNα2‐BCMAext‐mCherry in native conditions using Blue native PAGE (BN‐PAGE) since most members of the TNFRSF are preassociated in a ligand‐independent fashion [22, 23]. Nonetheless, BN‐PAGE revealed that IFNα2‐BCMAext‐GFP and IFNα2‐BCMAext‐mCherry were predominantly monomeric under native conditions (∼80%), with only a minor fraction (∼20%) forming trimers (Figure 2G). Because mCherry is intrinsically monomeric [24], these data strongly suggested that its limited trimerization reflects BCMA self‐association rather than fluorophore‐driven oligomerization.

### 2.4 High‐Binding Efficiency of Soluble BCMA Probes to CAR‐T Cells

Similar to IFNα2‐BCMAext‐GFP, IFNα2‐BCMAext‐mCherry exhibited a lower fluorescence intensity as compared with the commercial BCMA‐Fc‐biotin reagent (Figure 2H), ruling out the role of the GFP fluorochrome in the relatively low fluorescence intensity observed by flow cytometry. On the other hand, IFNα2‐BCMAext‐GFP and IFNα2‐BCMAext‐mCherry displayed markedly higher apparent binding efficiency for ide‐cel‐expressing Jurkat cells compared with the commercial BCMA‐Fc‐biotin reagent (Figure 2H). Indeed, dose‐response analyses revealed EC_50_ values of approximately 0.13 nM for both fluorescent BCMA constructs, compared with 2.8 nM for BCMA‐Fc‐biotin (Figure 2H). This difference may reflect conformational changes in BCMA‐Fc‐Biotin induced by Fc‐mediated dimerization since IgG1 Fc domain promotes the formation of parallel dimers through both disulfide bonds and noncovalent interactions [16]. Consistent with this notion, soluble Fc‐BCMA has been reported to bind both APRIL and BAFF, whereas γ‐secretase‐cleaved BCMA predominantly interacts with APRIL [4]. Differences in the length of the BCMA extracellular domain incorporated into each construct may also contribute to their distinct binding properties. While the BCMA‐Fc‐biotin reagent (Miltenyi Biotec) comprises amino acid residues 1–48 of the BCMA extracellular domain fused to the human IgG1 heavy‐chain Fc region and is secreted as an Fc‐BCMA fusion protein [25], our constructs contain the first 54 amino acids of human BCMA fused to a V5 tag and either eGFP or mCherry. Finally, the stronger signal intensity obtained with BCMA‐Fc‐biotin compared with the IFNα2‐BCMAext‐GFP and IFNα2‐BCMAext‐mCherry constructs may, at least partly, result from signal amplification during the secondary staining step using fluorochrome‐conjugated anti‐biotin antibodies or streptavidin.

### 2.5 Soluble BCMA Probes Stain CAR‐T Cells in Multiple Myeloma Patients

As IFNα2‐BCMAext‐GFP and IFNα2‐BCMAext‐mCherry exhibit comparable binding and detection sensitivity on CAR‐expressing Jurkat cells (Figure 2H) and IFNα2‐BCMAext‐mCherry undergoes a partial degradation (Figure 1H,F), we next kept IFNα2‐BCMAext‐GFP and assessed its ability to detect BCMA‐directed CAR‐T cells in the peripheral blood of patients with multiple myeloma (MM). Its performance was compared with that of two clinically validated CAR‐T‐cell detection reagents: BCMA‐Fc‐biotin followed by fluorescent anti‐biotin staining and an anti‐Whitlow/218 linker monoclonal antibody. IFNα2‐BCMAext‐GFP successfully identified circulating CAR‐T cells and, at a concentration of 2 mg/mL, yielded a detection profile comparable to that obtained with the anti‐Whitlow/218 linker mAb (Figure 2I), although the frequency of positive cells detected was slightly lower than that observed with the BCMA‐Fc‐biotin/fluorescent anti‐biotin staining strategy (Figure 2I). Notably, increasing concentrations of IFNα2‐BCMAext‐GFP resulted in enhanced staining intensity, indicating a dose‐dependent effect (Figure 2I). These findings suggest that further optimization of reagent concentration and purification may improve detection sensitivity.

We examined the correlation between IFNα2‐BCMAext‐GFP staining and the commercial detection reagents. A strong correlation was observed between IFNα2‐BCMAext‐GFP and both the anti‐Whitlow/218 linker and BCMA‐Fc‐biotin reagents (Figure 2J), supporting the reliability of IFNα2‐BCMAext‐GFP for CAR‐T‐cell detection. Finally, we observed that the addition of an AF488‐fused anti‐GFP mAb improved the quality of CAR‐T labeling in the blood of patients with MM (Figure 2K). Collectively, these results demonstrate that IFNα2‐BCMAext‐GFP represents a promising reagent for the detection of BCMA‐directed CAR‐T cells both in vitro and in peripheral blood samples from patients with MM.

### 2.6 Concluding Remarks

In this study, we developed soluble fluorescent BCMA‐based probes for the detection of anti‐BCMA CAR‐T cells. Beyond their immediate application for CAR‐T monitoring, these findings provide mechanistic insight into BCMA biosynthesis and establish a flexible platform for engineering soluble probes derived from other type III TNF receptor family members, including BAFF‐R, TACI, and XEDAR [26].

IFNα2‐BCMAext‐GFP construct specifically recognizes ide‐cel‐expressing cells and enables the detection of CAR‐T cells in peripheral blood samples from patients with multiple myeloma. Although the fluorescence intensity generated by the recombinant BCMA probes is lower than that obtained with the commercial BCMA‐Fc‐biotin reagent, the soluble BCMA constructs exhibit higher apparent binding efficiency toward ide‐cel‐expressing cells. These findings suggest that preservation of a more native BCMA conformation may enhance CAR recognition. Importantly, the probes provide a simple, one‐step staining strategy that may reduce costs and facilitate CAR‐T monitoring in research and translational laboratories. Accordingly, the production cost of IFNα2‐BCMA‐GFP and IFNα2‐BCMA‐mCherry is difficult to estimate precisely, as it includes consumables required for protein production and characterization (cell culture media, serum, antibodies for Western blotting and ELISA), as well as personnel costs. Nevertheless, once the production protocol has been established, these reagents represent a cost‐effective alternative for academic laboratories compared with commercially available CAR detection reagents. For example, the BCMA‐Fc‐Biot detection reagent (30 tests; €990; #130‐126‐090), together with the required anti‐biotin antibody (30 tests; €73; #130‐111‐069), or the anti‐Whitlow reagent (30 tests; €369; #130‐137‐251), remains substantially more expensive on a per‐test basis. Production of IFNα2‐BCMA‐GFP and IFNα2‐BCMA‐mCherry only requires standard laboratory procedures, including calcium phosphate transfection of HEK293T cells, ultracentrifugation of conditioned medium, and supernatant concentration using centrifugal filter devices. A single production run using ten culture dishes (10‐cm dishes seeded with 1 × 10^6^ cells) will typically yield 1 to 5 mL of IFNα2‐BCMA‐GFP or IFNα2‐BCMA‐mCherry at a concentration of approximately 100 µg/mL. If we use 50 µL of reagent diluted to 10 µg/mL per condition to detect CAR‐T cells (0.5 µg per test), a single production batch will be sufficient for approximately 200 to 1000 staining reactions. Consequently, although the exact production cost depends on local laboratory expenses, the cost per assay is expected to be substantially lower than that of commercially available anti‐Whitlow or BCMA‐Fc‐Biot detection reagents.

Collectively, our results establish soluble fluorescent BCMA proteins as promising and accessible tools for the detection of BCMA‐directed CAR‐T cells.

### 2.7 Study Limitations

We identify different limitations in this study. First, the study was conducted using a limited number of patient samples, which restricts the assessment of assay performance across diverse clinical settings. Second, the recombinant probes are evaluated primarily with ide‐cel CAR‐T cells; therefore, their applicability to other BCMA‐targeting CAR constructs remains to be determined. Third, despite their high binding efficiency, the fluorescent BCMA probes generate lower fluorescence signals than the commercially available BCMA‐Fc‐biotin reagent, indicating that further optimization of fluorophore brightness, protein purification, or signal amplification strategies may improve sensitivity. Finally, the molecular mechanism underlying the enhanced binding properties of the soluble BCMA constructs compared with BCMA‐Fc reagent will require additional structural and biophysical studies.

## 3 Material and Methods

### 3.1 Patients Samples

Patients were enrolled at the University Hospital of Saint‐Etienne (France) in 2026. This study was approved by the local ethics committee (IRBN 89–2026/CHUSTE) and was conducted in accordance with the 1964 Helsinki Declaration and its subsequent amendments. We included all MM patients treated with anti‐BCMA CART cells idecabtagene vicleucel at the time of the study, with the following exclusion criteria: (i) being under 18 years of age, (ii) patients expressing their opposition to participating in the study, and (iii) no available samples or sample volume. The flow cytometry measurements were performed at the University Hospital of Saint‐Etienne.

### 3.2 Reagents

Fetal Bovine Serum (#A5256801), Opti‐MEM medium (#51985‐026), Alexa Fluor 647‐conjugated goat anti‐Mouse IgG1 (H+L) (#A21240), Alexa Fluor 488‐conjugated donkey anti‐mouse IgG (H+L) (#A32766), Lipofectamine 2000 (#11668019), Dynabeads MyOne Streptavidin T1 (#65601), Streptavidin–HRP (#3130656), Streptavidin‐Alexa Fluor 488 (#S11223) were from Invitrogen/Thermo Fisher Scientific (Carlsbad, CA, and Waltham, MA, USA). Amicon Ultra‐15 Centrifugal Filter Units (10 kDa MW) (#UFC901008) came from Millipore (Burlington, MA, USA). DMEM high glucose (#MS02NW1009) and bovine serum albumin (#PAO22AK1F9) came from BioSeră (Nuaillé, France). RPMI 1640 (#MS02Q8100A) was from BioWest (Nuaillé, France). Dulbecco's PBS, without Ca^2^^+^ and Mg^2^^+^ (#CS1PBS01‐01), was purchased from Eurobio Scientific (Les Ulis, France). PE‐conjugated anti‐human BCMA/TNFRSF17 monoclonal antibody (#FAB193P) and goat anti‐human BCMA/TNFRSF17 polyclonal antibody (#AF193) were purchased from R&D Systems (Minneapolis, MN, USA). Biotinylated BCMA CAR Detection Reagent (BCMA‐Fc‐biotin) (#130‐126‐090), APC‐conjugated anti‐biotin (#130‐111‐069), and Whitlow/218 Linker‐PE (#130‐137‐251) were from Miltenyi Biotec (Bergisch Gladbach, Germany). AF488‐conjugated anti‐GFP monoclonal antibody (#ab225314) came from Abcam (Cambridge, UK). ViaKrome 808 Fixable Viability Dye (#C36628), ECD‐conjugated anti‐human CD45 monoclonal antibody (#A07784), PB‐conjugated anti‐human CD3 monoclonal antibody (#B49204), and VersaLyse Lysing Solution (#A09777) came from Beckman Coulter Life Sciences (Brea, CA, USA). Purified Mouse Anti‐GM130 (clone 35/GM130; #610823) was from BD Biosciences (Franklin Lakes, NJ, USA). Anti‐β‐actin mAb (clone AC‐15, #A5441) came from Sigma‐Aldrich (St. Louis, MO, USA). Goat TrueBlot ULTRA (Anti‐Goat IgG‐HRP; #18‐8814‐31) came from Rockland Immunochemicals (Limerick, PA, USA). Mouse anti‐V5 mAb (clone SV5‐Pk1, #MCA1360) was purchased from Bio‐Rad (Hercules, CA, USA). Goat anti‐mouse IgG2a‐HRP (#1080‐05) and mouse anti‐rabbit IgG‐HRP (#1070‐05) came from SouthernBiotech (Birmingham, AL, USA).

### 3.3 Vectors

Wild‐type BCMA (Q02223‐1) without a stop codon was fused in frame with a V5 tag (GKPIPNPLLGLDST) and inserted between XhoI/BamHI in the pEGFP‐N1 vector to generate BCMA‐GFP. Similarly, BCMAext (amino acid residues 1–54) was fused to a V5 tag and inserted between XhoI/BamHI in pEGFP‐N1 to generate BCMAext‐GFP. For the IFNα2‐BCMAext‐GFP and IFNα2‐BCMAext‐mCherry constructs, the signal peptide of human IFNα2 (MALTFALLVALLVLSCKSSCSVG) described in [17] was inserted at the 5’ end of the BCMAext sequence (amino acid residues 2 to 54), in which the initial methionine was eliminated. This gene was inserted between EcoRI/BamHI into the pLVX‐IRES‐puro vector (Clontech) in frame with V5 tag and eGFP or mCherry sequences. For each construct, a Kozak sequence (GCGACC) was inserted before the initiating codon ATG. Constructs were synthesized and cloned by Genecust (Chalmont, France), and sequences were verified by DNA sequencing on both strands.

### 3.4 Production of BCMA‐GFP and ‐mCherry Reagents

HEK/293T cells were seeded at 1.10^6^ cells per 10 cm dish in DMEM supplemented with 10% fetal bovine serum (FBS). The calcium phosphate transfection solution was prepared by mixing 430 µL sterile water, 70 µL CaCl_2_ (2 M), and 3 µg plasmid DNA, followed by the addition of 500 µL HEPES‐buffered saline (55 mM HEPES, 1.5 mM Na_2_HPO_4_, and 274 mM NaCl at pH 7.05). The suspension was mixed by pipetting and incubated for 10 min at room temperature, then added dropwise to the cells. After 24 h, the medium was replaced by fresh OPTI‐MEM, and supernatants containing secreted recombinant BCMA were collected at day 7. Supernatants were ultracentrifuged for 1 h (100,000*g*) to eliminate exosomes and then concentrated using centrifugal filters (Amicon Ultra‐15, Ultracel‐10K). Supernatants were sterilized using 0.22 µM filters and stored at 4°C. Soluble BCMA was dosed by ELISA.

### 3.5 BCMA ELISA

Soluble BCMA constructs were dosed by ELISA following the manufacturer's instructions (#AB263875, Abcam).

### 3.6 Lentivirus Production

HEK/293T cells (5 × 10^6^ cells/mL) were seeded in 10 cm dishes, and after 24 h, the lentiviral vector (20 µg), packaging plasmid psPAX2 (5 µg), and envelope plasmid pMD2.G (5 µg) were transfected using lipofectamine (Life Technologies, Carlsbad, USA) in a final volume of 10 mL. After 24 h, the transfection media were replaced with fresh DMEM containing 10% FBS. Viral supernatant was collected at 72 h posttransfection, centrifuged at 3000*g* for 10 min to remove cell debris, filtered (0.45 µm), aliquoted, and stored at −80°C.

Transduction was performed by adding 1 mL of viral production dropwise onto 70,000 HEK/293T or 250,000 Jurkat cells seeded onto 12‐well plates in a final volume of 2 mL. Infection was enhanced with 2 µg/mL polybrene and centrifugation at 600*g* for 45 min at 37°C. After overnight incubation, the transduction media were replaced with fresh DMEM supplemented with 10% FBS, then re‐incubated for 72 h before harvest.

### 3.7 Flow Cytometry

CAR ide‐cel surface expression was determined by cell staining with BCMA‐Fc‐biotin or IFNα2‐BCMAext‐GFP or ‐mCherry. Briefly, cells (1.10^6^) were washed in PBS and coated with PBS containing 1% bovine serum albumin (BSA) on ice for 10 min. Cells were then incubated with BCMA‐Fc‐biotin, IFNα2‐BCMAext‐GFP, or IFNα2‐BCMAext‐mCherry constructs for 10 min on ice. For BCMA‐Fc‐biotin, BCMA was revealed by incubating cells with Alexa488‐conjugated streptavidin for 10 min on ice. Then, cells were washed in PBS supplemented with 1% BSA and incubated with Viakrome 808 for 5 min in the dark and on ice before a final wash. Cells were finally resuspended in 200 µL of PBS with 1% BSA and analyzed by flow cytometry using a CytoFLEX LX flow cytometer (Beckman Coulter Life Sciences). To decrease antibody internalization and background, all steps were performed at 4°C.

### 3.8 CAR‐T Cell Monitoring in Patient Blood

100 µL of whole blood was washed with PBS. Cells were then incubated for 10 min with anti‐CD45‐ECD and anti‐CD3‐PB together with either biotinylated BCMA CAR Detection Reagent (BCMA‐Fc‐biotin), Whitlow/218 Linker‐PE, or IFNα2‐BCMAext‐GFP. Cells were subsequently washed in PBS. For samples stained with BCMA‐Fc‐biotin, bound reagent was detected by incubation with APC‐conjugated anti‐biotin for 10 min, followed by a PBS wash. For samples stained with IFNα2‐BCMAext‐GFP, GFP fluorescence was analyzed directly or, when indicated, amplified by incubation with AF488‐conjugated anti‐GFP for 10 min, followed by a PBS wash. Red blood cell lysis was then performed by incubating the cell pellet with 1 mL of VersaLyse Lysing Solution for 10 min, followed by a PBS wash. Cells were finally resuspended in 300 µL of PBS supplemented with 1% BSA and 0.8% EDTA. Samples were immediately run on a DxFLEX flow cytometer, and data were analyzed using CytExpert software (Beckman Coulter Life Sciences).

### 3.9 Immunoblotting

Transfected cells were washed once with cold PBS and lysed for 30 min on ice using a RIPA buffer supplemented with protease and phosphatase inhibitor cocktail (Sigma). Protein concentration was determined by the bicinchoninic acid method (Pierce, Rockford, IL, USA) according to the manufacturer's protocol. A total of 60 µg of protein was loaded per lane, resolved by 12% SDS‐PAGE gel (Bio‐Rad Laboratories), and transferred to a PVDF membrane (GE Healthcare, Buckinghamshire, UK). Nonspecific binding sites were blocked by incubating membranes for 1 h with TBST‐milk (50 mM Tris, 160 mM NaCl, 0.1% (v/v) Tween 20, and 5% (w/v) dried skimmed milk at pH 7.4) and later incubated overnight at 4°C with the corresponding primary antibody. The next day, membranes were washed thrice in PBS‐Tween 0.1% for 10 min, incubated for 1 h with secondary antibodies, and washed again as mentioned above. For visualization of bound primary antibodies, membranes were revealed using an enhanced chemiluminescence detection system (#WBLUF0500, Immobilon Forte Western HRP Substrate, Millipore) on Fusion Spectra Imaging System (Vilber Lourmat).

### 3.10 IFNα2‐BCMAext‐GFP and mCherry Immunoprecipitation and Glycosylation Analysis

One ml of SN IFNα2‐BCMAext‐GFP and IFNα2‐BCMAext‐mCherry were subjected to immunoprecipitation using 60 µl of anti‐V5 magnetic beads (#SAE0203, Sigma Aldrich, St. Louis, MO, USA). The IP was incubated overnight at 4°C on a rotary shaker. Beads were washed three times with ice‐cold PBS and resuspended in 60 µL of PBS, and 50 µL was used for deglycosylation experiments. N‐glycan removal was performed using PNGase F glycan cleavage kit (#A39245, Gibco). O‐glycan removal was performed using O‐glycosidase (#P0733, New England Biolabs) and α2‐3,6,8,9 neuraminidase A (#P0722, New England Biolabs). O‐ and N‐glycans were removed simultaneously using Protein deglycosylation Mix II (#P6044, New England Biolabs). Deglycosylation was performed according to the manufacturer's instructions. Briefly, 12.5 µL of beads were treated or untreated with enzymes (for 1 h at 50°C for PNGase F, for 4 h at 37°C for O‐glycosidase/neuraminidase A and Deglycosylation Mix II). Beads were washed three times with PBS and resuspended in 20 µL of Laemmli sample buffer under reducing conditions. Beads were removed using a magnet, and proteins were collected and resolved in a 12% SDS‐PAGE for immunoblot analysis.

### 3.11 BN‐PAGE

Protein supernatants were subjected to native electrophoresis on NativePAGE Novex Bis‐Tris Gel system following manufacturer's instructions (ThermoFisher). Briefly, supernatants were prepared using the NativePAGE sample Prep Kit (ThermoFisher, #BN2008) and loaded on 4%–16% Bis‐Tris NativePAGE gels (ThermoFisher, #BN1002BOX). Proteins were transferred to PVDF membranes (Invitrolon PVDF Filter Paper Sandwich, #LC2005) using NuPAGE Transfer Buffer (ThermoFisher, #NP0006).

### 3.12 Confocal Analyses

Transfected HEK/293T cells expressing GFP‐ and mCherry‐coupled BCMA constructs were grown on eight‐well polycycloalcanes (PCA) slides (Sarstedt) for 16 h. Cells were fixed with 4% PFA for 15 min at room temperature. The Golgi apparatus was stained using mouse IgG1 anti‐GM130 (# 610823, BD Biosciences) diluted in BSA1%‐saponin 0.05% and incubated overnight at 4°C. Primary antibody was detected with an AlexaFluor 647‐coupled goat anti‐mouse IgG antibody or AlexaFluor 488‐coupled donkey anti‐mouse IgG antibody in BSA 1%‐saponin 0.05% and incubated 1 h at 37°C. Cell nuclei were stained with DAPI. Slides were mounted with Mowiol medium and conserved at 4°C, protected from light. Fluorescence acquisitions were taken using the LSM 880 confocal microscope (Zeiss). Images were taken using a 40× objective.

## Author Contributions

L.P.V.N., C.O., C.C., L.W., F.K., A.B., C.P., and M.M. conceived and conducted research. L.W., A.B., A.H., J‐M.G., X.L., E.C., and M.R. provided reagents and study patients. M.R. and P.L. supervised the research activities. P.L. prepared and wrote the manuscript.

## Ethics Statement for Human and/or Animal Studies

The manuscript does not contain experiments using animals. The manuscript does contain human samples, and we included local Ethical Committee approval received for the study.

## Patient Consent Statement

We obtained informed consent from all participating subjects.

## Permission to Reproduce Material from Other Sources

The authors have nothing to report.

## Conflicts of Interest

The authors declare no conflicts of interest.

## Data Availability

The data that support the findings of this study are available from the corresponding author upon reasonable request.
